# Supplementary material for: Dispersion Performances of Naphthalimides Doped in Dual Temperature- and pH-Sensitive Poly (N-Isopropylacrylamide-co-acrylic Acid) Shell Assembled with Vinyl-Modified Mesoporous SiO2 Core for Fluorescence Cell Imaging
Source: Polymers (Basel). 2023 May 17;15(10):2339. doi: 10.3390/polym15102339 (PMC10222162; doi:10.3390/polym15102339)
Supplement: Supplementary file 1 [file polymers-15-02339-s001.zip › polymers-2209743-supplementary.pdf]

Dispersion performances of naphthalimides doped in dual temperature- and pH-sensitive poly (N-isopropylacrylamide-co-acrylic acid) shell assembled with vinyl-modified mesoporous SiO_2 core for fluorescence cell imaging

Xiaohuan Xu, Xiaoli Wang, Xueqing Cui, Bingying Jia, Bang Xu*, Jihong Sun*

Beijing Key Laboratory for Green Catalysis and Separation, Department of Chemical Engineering, Beijing University of Technology, Beijing 100124, China

Electronic Supplementary Information

Figure S1. Regression equation and HPLC standard curve of IBU concentration in absolute ethanol.

Figure S2. Regression equations and the standard curves of IBU concentration in PBS at (A) pH 2.0 and (B) pH 7.4.

Figure S3. Small angle XRD patterns (insert: wide angel) (A) of before and after IBU loading samples, (a) PAN@BMMs-II-0.1, (b) PAN@BMMs-mix-0.1, (c) I/PAN@BMMs-II-0.1, and (d) I/PAN@BMMs-mix-0.1. N_2 adsorption-desorption isotherms (right: the offset values were remarked in the Y-axis; insert: pore size distribution) (B) of (a) BMMs, (b) M-BMMs, (c) PAN@BMMs-I-0.05, (d) PAN@BMMs-I-0.1, (e) PAN@BMMs-I-1, (f) I/PAN@BMMs-I-0.05, (g) I/PAN@BMMs-I-0.1, (h) I/PAN@BMMs-I-1, and (C) of (a) PAN@BMMs-II-0.1, (b) PAN@BMMs-mix-0.1, (c) I/PAN@BMMs-II-0.1, (d) I/PAN@BMMs-mix-0.1.

Figure S4. FT-IR spectra (A) (a) BMMs, (b) M-BMMs, (c) P(NIPAM-co-AA), (d) AN, (e) PAN@BMMs-I-0.1, (f) I/PAN@BMMs-I-0.1, and (g) IBU. At the region of 2000-400 cm^{-1} (B) (a) PAN@BMMs-I-0.05, (b) PAN@BMMs-I-1, (c) PAN@BMMs-II-0.1, and (d) PAN@BMMs-mix-0.1.

Figure S5. XPS survey spectra (A), N1s spectra (B), and C1s spectra (C) of (a)

P(NIPAM-co-AA)@BMMs and (b) PAN@BMMs-I-0.1.

Figure S6. Fluorescence emission spectra of AN (2×10^{-3} mol/L) in various solvents, (a) PE, (b) 1,4-Dioxane, (c) EtOH, (d) MeCN, and (e) DMSO.

Figure S7. TGA curves of (a) BMMs, (b) M-BMMs, (c) P(NIPAM-co-AA), (d) PAN@BMMs-I-0.05, (e) PAN@BMMs-I-0.1, (f) PAN@BMMs-I-1, (g) PAN@BMMs-II-0.1, (h) PAN@BMMs-mix-0.1, and (i) AN.

Figure S8. Size distribution of BMMs nanomaterial.

Figure S9. Swelling time-depended fluorescent spectra of (A) PAN@BMMs-I-0.05, (B) PAN@BMMs-I-0.1, (C) PAN@BMMs-I-1, (D) PAN@BMMs-II-0.1, and (E) PAN@BMMs-mix-0.1, at (a) pH 7.4 25 ºC and (b) pH 2.0 37 ºC, Notes: S represents the solid sample and L is the filtrated solution.

Figure S10. Ln-Ln plots originating from the SAXS patterns of PAN@BMMs-I-0.05 (A) in pH 7.4 at 25 ºC and (B) in pH 2.0 at 37 ºC over time, PAN@BMMs-I-1 (C) in pH 7.4 at 25 ºC and (D) in pH 2.0 at 37 ºC over time. Their corresponding *P(r)-r* charts were inserted. (a) 1 h, (b) 3 h, (c) 8 h, (d) 12 h, (e) 24 h. Notes: The linear ranges with a higher coefficient ($R^2 > 0.99$) were $-2.20 < lnq < -1.52$. The vertical offset values were presented in the right Y-axis

Figure S11. Fluorescent spectra of (A) I/PAN@BMMs-I-0.05, (B) I/PAN@BMMs-I-0.1, (C) I/PAN@BMMs-I-1, (D) I/PAN@BMMs-II-0.1, (E) I/PAN@BMMs-mix-0.1 during the IBU release process (a) in pH 2.0 at 37 ºC and (b) in pH 7.4 at 25 ºC. Notes: S represents the solid sample and L is the filtrated solution.

Figure S12. Time-dependent CLSM images (6 h, 12 h, 24 h) of HeLa cells incubated as controlled samples, scale bars were 10 μm.

Table S1. The fluorescence decay data of all related samples.

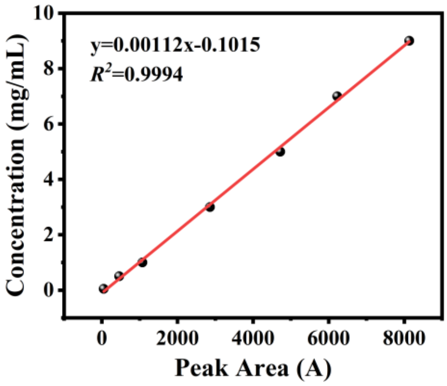

Figure S1. Regression equation and HPLC standard curve of IBU concentration in absolute ethanol.

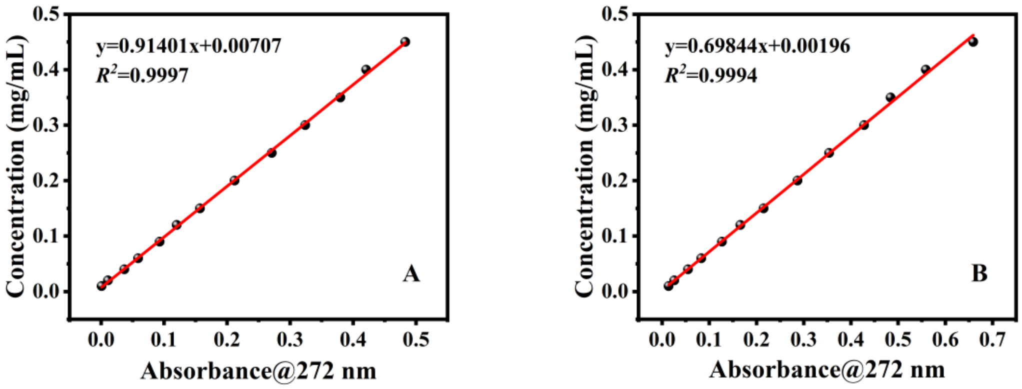

Figure S2. Regression equations and the standard curves of IBU concentration in PBS at (A) pH 2.0 and (B) pH 7.4.

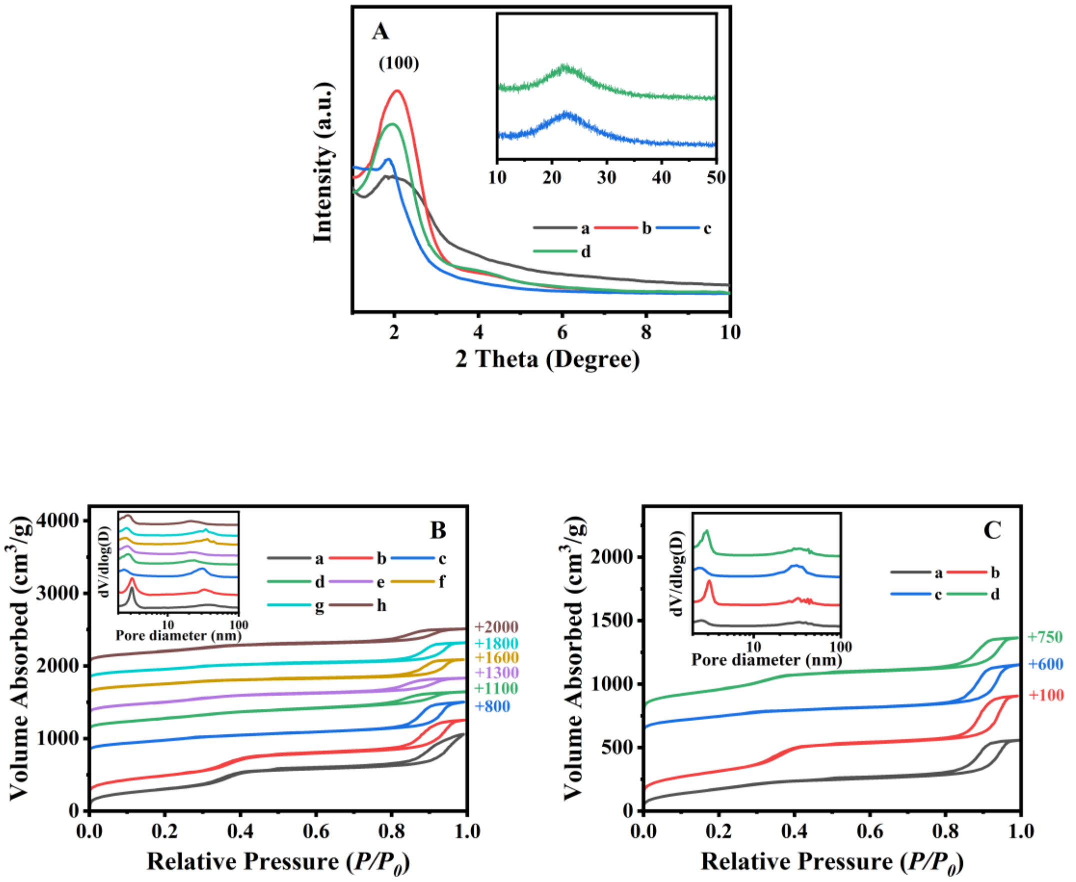

Figure S3. Small angle XRD patterns (insert: wide angel) (A) of before and after IBU loading samples, (a) PAN@BMMs-II-0.1, (b) PAN@BMMs-mix-0.1, (c) I/PAN@BMMs-II-0.1, and (d) I/PAN@BMMs-mix-0.1. N_2 adsorption-desorption isotherms (right: the offset values were remarked in the Y-axis; insert: pore size distribution) (B) of (a) BMMs, (b) M-BMMs, (c) PAN@BMMs-I-0.05, (d) PAN@BMMs-I-0.1, (e) PAN@BMMs-I-1, (f) I/PAN@BMMs-I-0.05, (g) I/PAN@BMMs-I-0.1, (h) I/PAN@BMMs-I-1, and (C) of (a) PAN@BMMs-II-0.1, (b) PAN@BMMs-mix-0.1, (c) I/PAN@BMMs-II-0.1, (d) I/PAN@BMMs-mix-0.1.

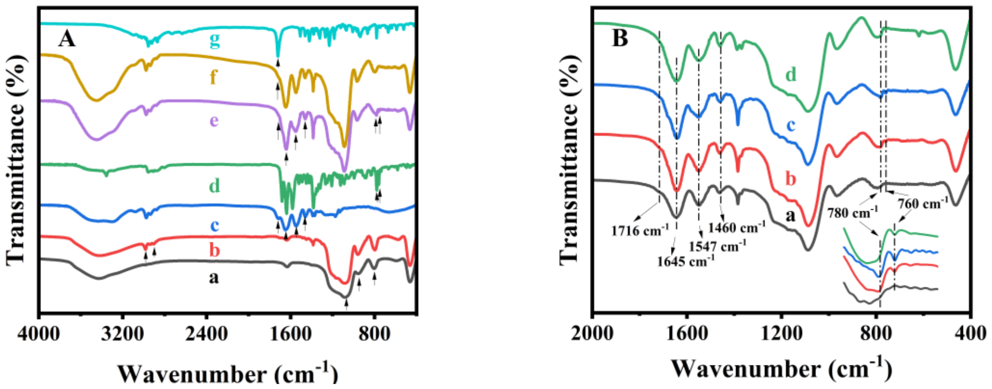

Figure S4. FT-IR spectra (A) (a) BMMs, (b) M-BMMs, (c) P(NIPAM-co-AA), (d) AN, (e) PAN@BMMs-I-0.1, (f) I/PAN@BMMs-I-0.1, and (g) IBU. At the region of 2000-400 cm^{-1} (B) (a) PAN@BMMs-I-0.05, (b) PAN@BMMs-I-1, (c) PAN@BMMs-II-0.1, and (d) PAN@BMMs-mix-0.1.

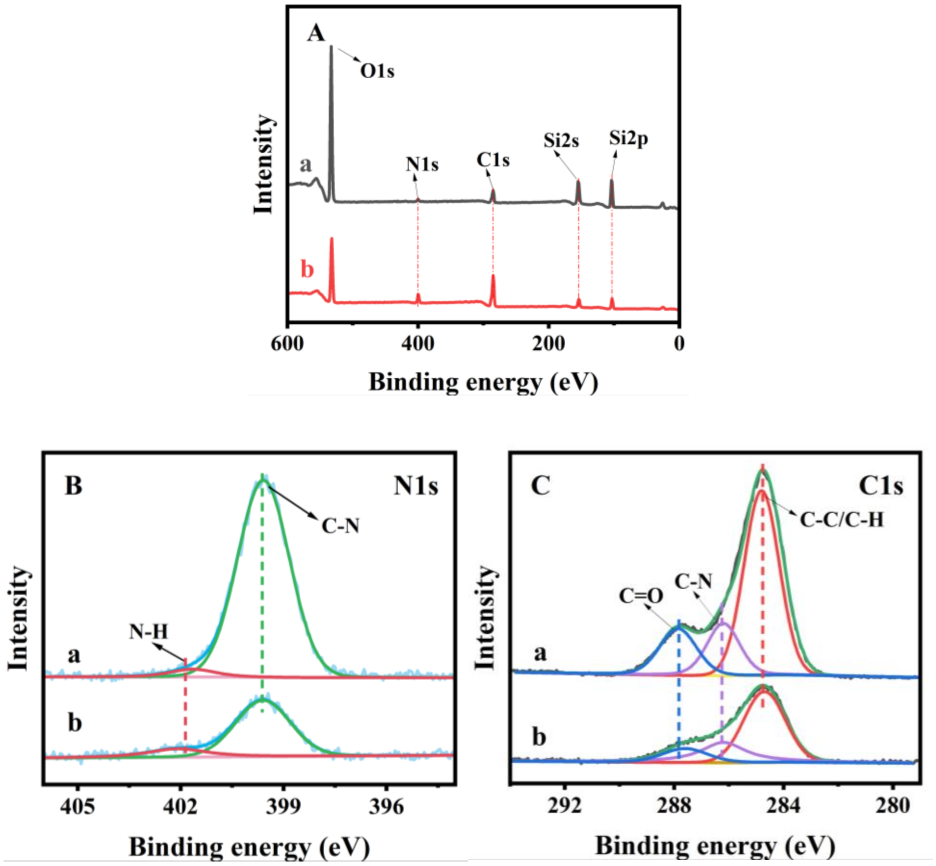

Figure S5. XPS survey spectra (A), N1s spectra (B), and C1s spectra (C) of (a) P(NIPAM-co-AA)@BMMs and (b) PAN@BMMs-I-0.1.

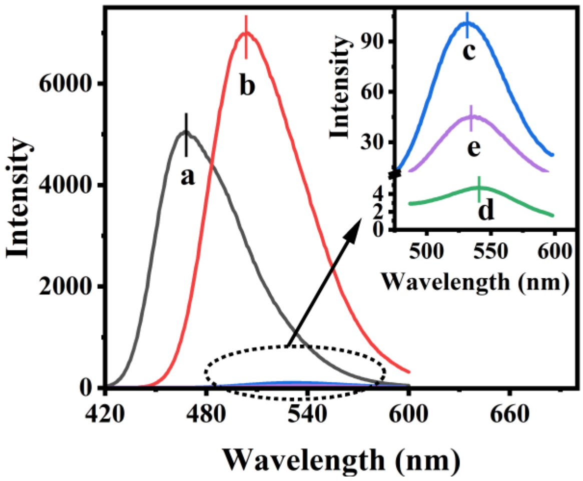

Figure S6. Fluorescence emission spectra of AN (2×10^{-3} mol/L) in various solvents, (a) PE, (b) 1,4-Dioxane, (c) EtOH, (d) MeCN, and (e) DMSO.

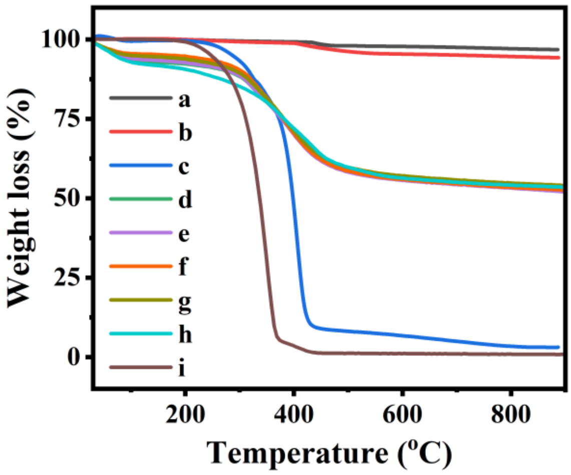

Figure S7. TGA curves of (a) BMMs, (b) M-BMMs, (c) P(NIPAM-co-AA), (d) PAN@BMMs-I-0.05, (e) PAN@BMMs-I-0.1, (f) PAN@BMMs-I-1, (g) PAN@BMMs-II-0.1, (h) PAN@BMMs-mix-0.1, and (i) AN.

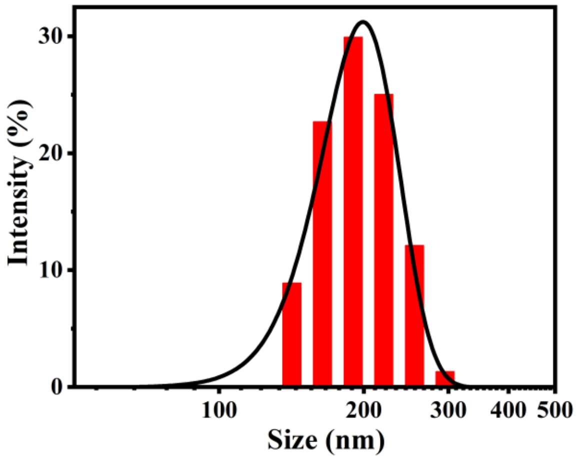

Figure S8. Particle size distribution of the synthesized BMMs.

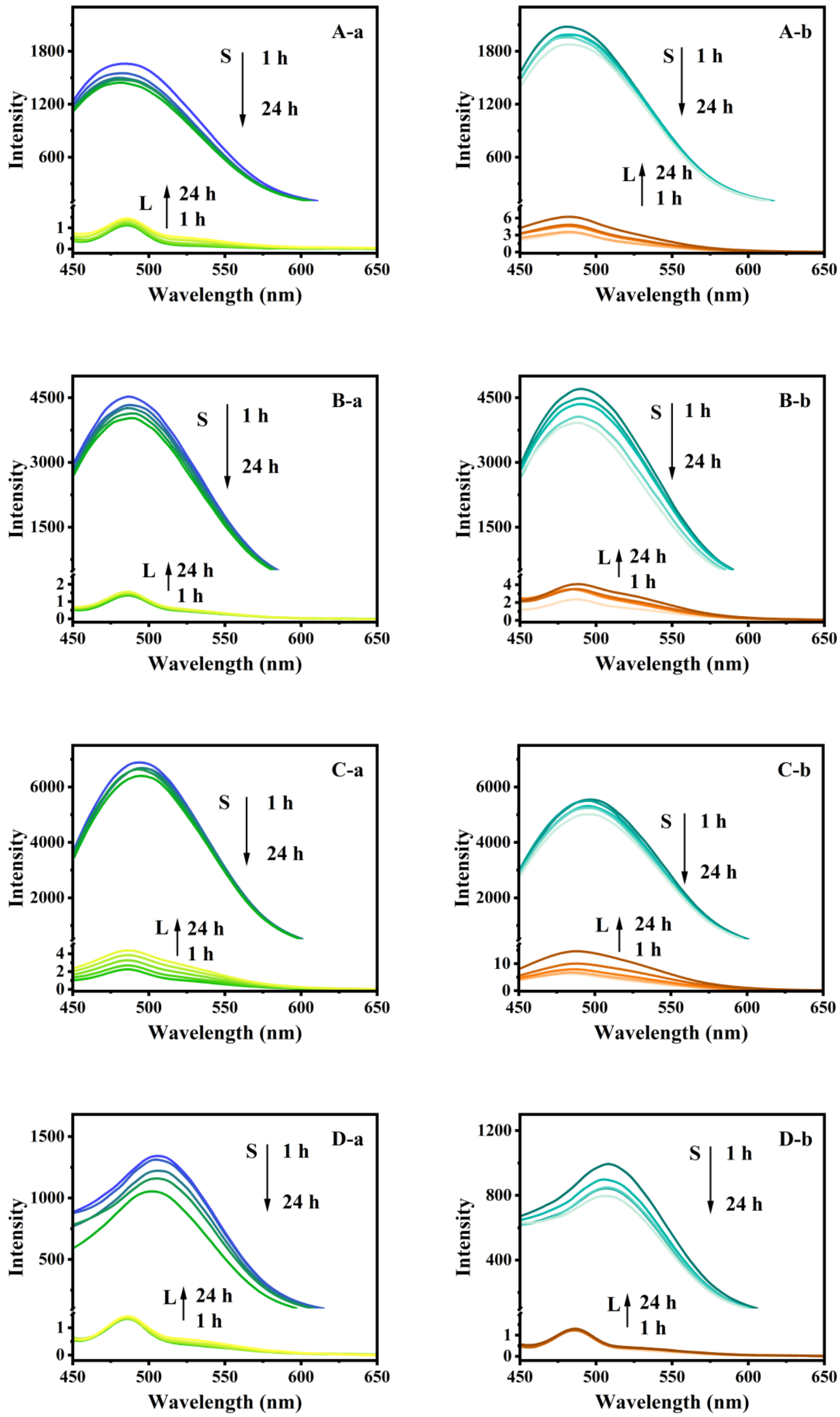
A-a
S 1 h
24 h
L 24 h
1 h
Intensity
Wavelength (nm)
A-b
S 1 h
24 h
L 24 h
1 h
Intensity
Wavelength (nm)
B-a
S 1 h
24 h
L 24 h
1 h
Intensity
Wavelength (nm)
B-b
S 1 h
24 h
L 24 h
1 h
Intensity
Wavelength (nm)
C-a
S 1 h
24 h
L 24 h
1 h
Intensity
Wavelength (nm)
C-b
S 1 h
24 h
L 24 h
1 h
Intensity
Wavelength (nm)
D-a
S 1 h
24 h
L 24 h
1 h
Intensity
Wavelength (nm)
D-b
S 1 h
24 h
L 24 h
1 h
Intensity
Wavelength (nm)

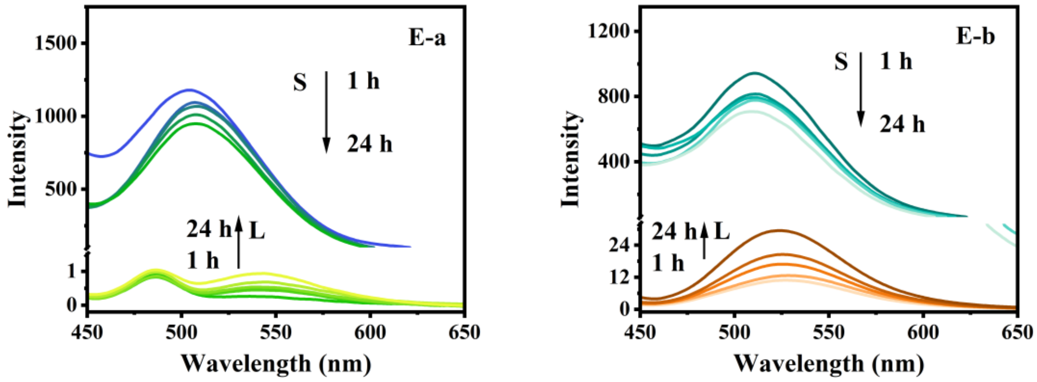

Figure S9. Swelling time-depended fluorescent spectra of (A) PAN@BMMs-I-0.05, (B) PAN@BMMs-I-0.1, (C) PAN@BMMs-I-1, (D) PAN@BMMs-II-0.1, and (E) PAN@BMMs-mix-0.1, at (a) pH 7.4 25 ºC and (b) pH 2.0 37 ºC, Notes: S represents the solid sample and L is the filtrated solution.

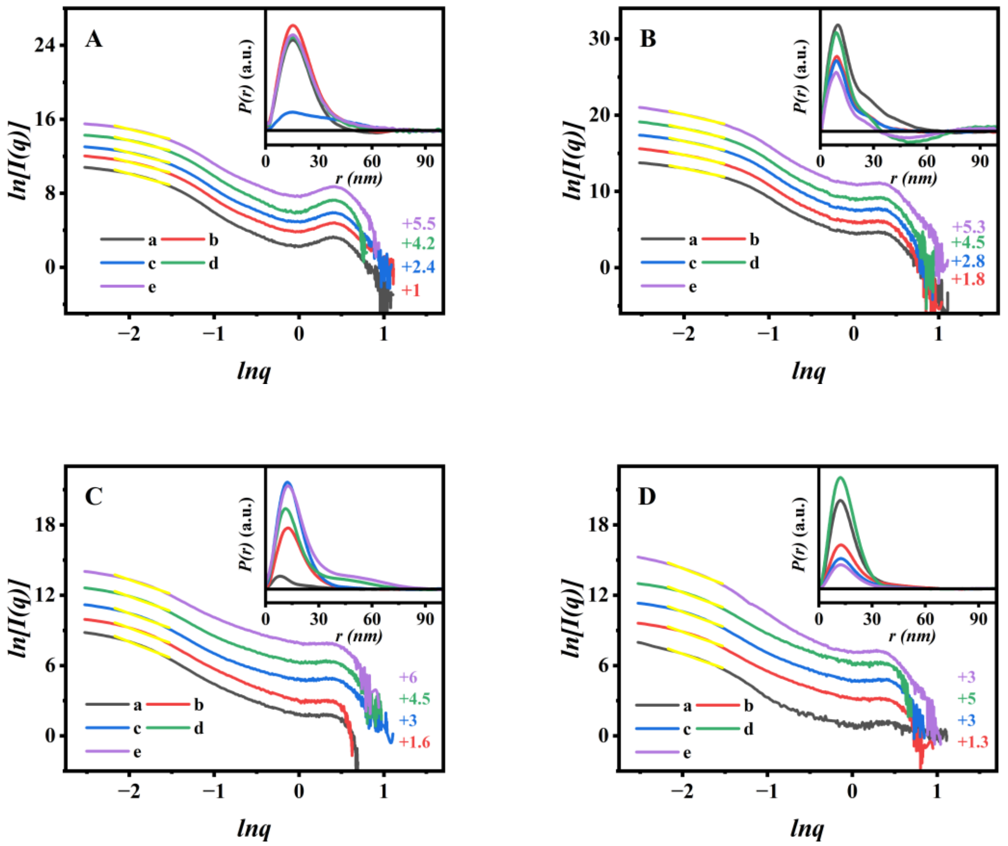

Figure S10. Ln-Ln plots originating from the SAXS patterns of PAN@BMMs-I-0.05 (A) in pH 7.4 at 25 ºC and (B) in pH 2.0 at 37 ºC over time, PAN@BMMs-I-1 (C) in pH 7.4 at 25 ºC and (D) in pH 2.0 at 37 ºC over time. Their corresponding *P(r)-r* charts were inserted. (a) 1 h, (b) 3 h, (c) 8 h, (d) 12 h, (e) 24 h. Notes: The linear ranges with a higher coefficient ($R^2 > 0.99$) were $-2.20 < lnq < -1.52$. The vertical offset values were presented in the right Y-axis.

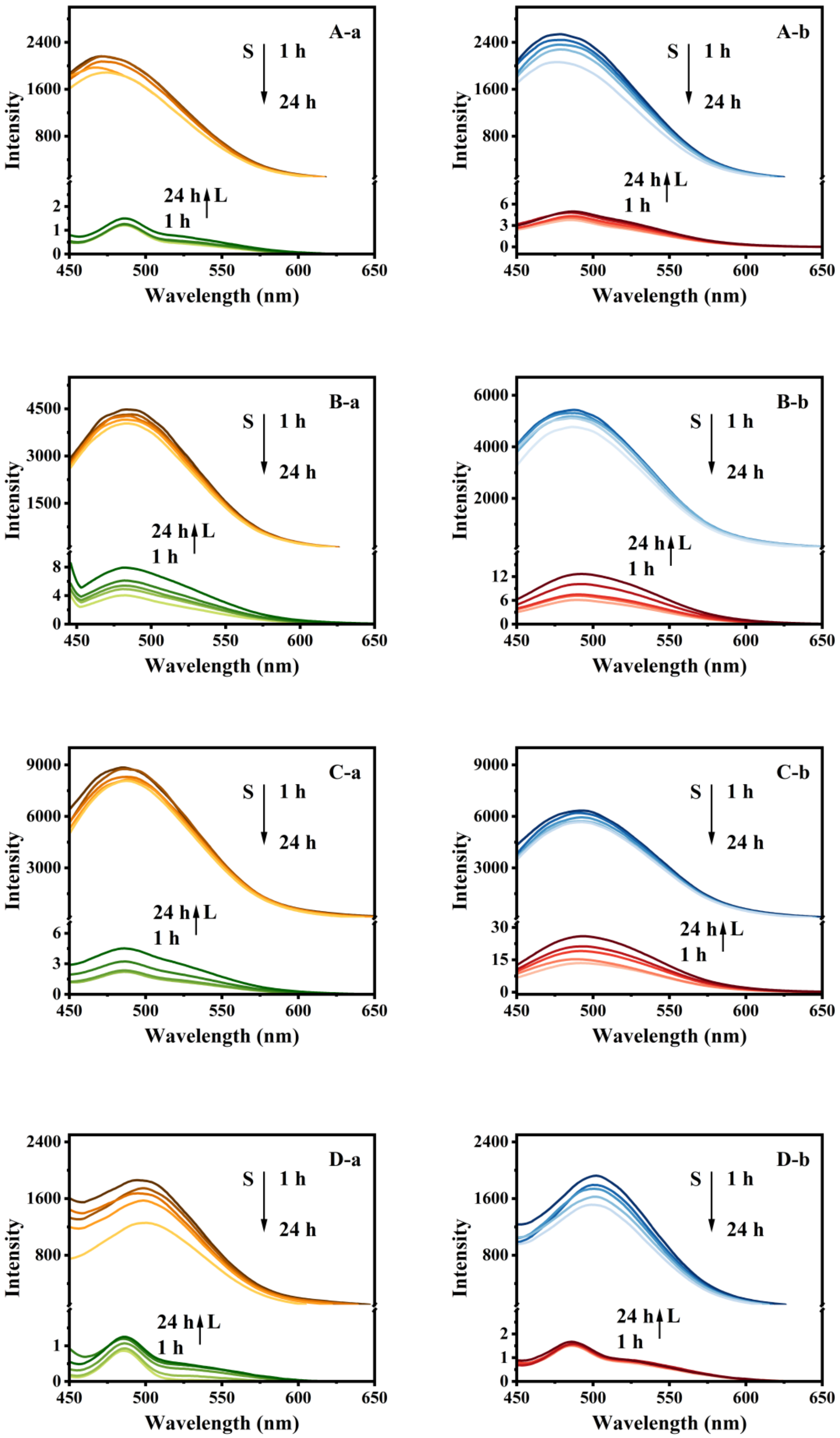
A-a
A-b
B-a
B-b
C-a
C-b
D-a
D-b
S
1 h
24 h
L
Intensity
Wavelength (nm)

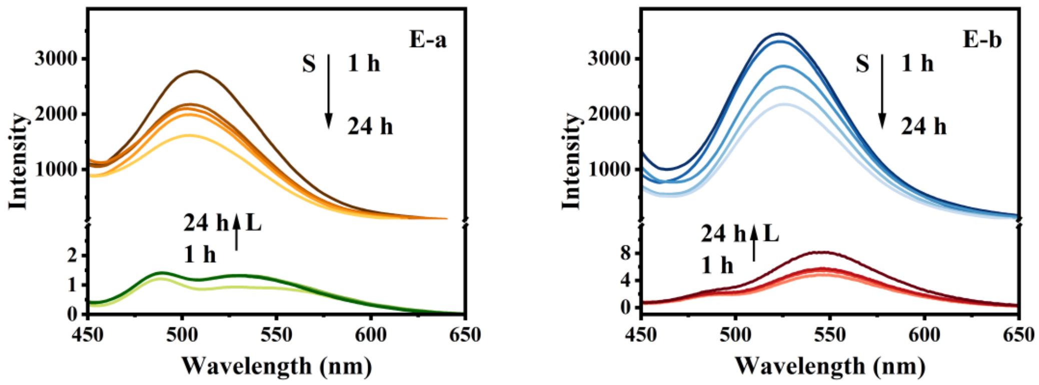

Figure S11. Fluorescent spectra of (A) I/PAN@BMMs-I-0.05, (B) I/PAN@BMMs-I-0.1, (C) I/PAN@BMMs-I-1, (D) I/PAN@BMMs-II-0.1, (E) I/PAN@BMMs-mix-0.1 during the IBU release process (a) in pH 2.0 at 37 ºC and (b) in pH 7.4 at 25 ºC. Notes: S represents the solid sample and L is the filtrated solution.

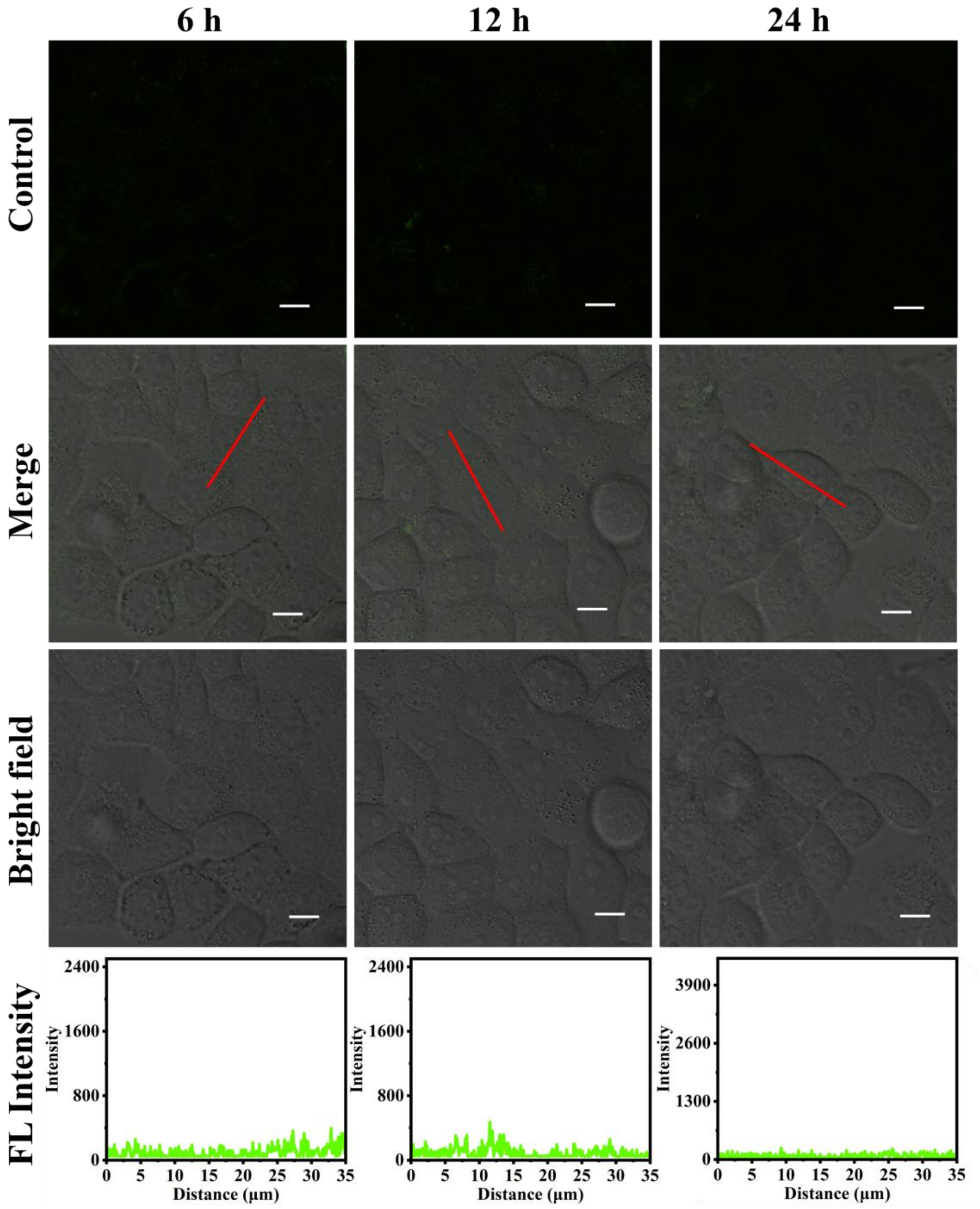

Figure S12. Time-dependent CLSM images (6 h, 12 h, 24 h) of HeLa cells incubated as the controlled samples, scale bars were 10 μm.

Table S1. The fluorescence decay data of all related samples

Samples	fluorecsence lifetime/ns				χ^2	*A*
	τ_1/ns (B_1/%)		τ_2/ns (B_2/%)			
AN	3.78	40.74	12.27	59.26	1.0860	1.1802
PNA@BMMs-I-0.05	3.35	39.77	10.50	60.23	1.0613	1.4684
PNA@BMMs-I-0.1	3.59	40.52	10.62	59.48	1.0550	1.6255
PNA@BMMs-I-1	3.89	36.17	10.92	63.83	1.0566	1.6804
PNA@BMMs-II-0.1	2.64	40.04	8.56	59.96	1.0433	1.3830
PNA@BMMs-mix-0.1	3.53	24.12	9.00	75.88	1.0787	2.9830
